# Traumatic Events and Substance Use Disorders in Adolescents

**DOI:** 10.3389/fpsyt.2020.00559

**Published:** 2020-06-18

**Authors:** Lukas A. Basedow, Sören Kuitunen-Paul, Veit Roessner, Yulia Golub

**Affiliations:** Department of Child and Adolescent Psychiatry, Faculty of Medicine, TU Dresden, Dresden, Germany

**Keywords:** teenager, trauma, addiction, self-medication, traumatic experiences

## Abstract

**Objectives:**

Adolescents with substance use disorders (SUD) frequently report traumatic events (TEs) and symptoms of post-traumatic stress disorder (PTSD). This study aimed to assess whether lifetime prevalence rates of TEs and PTSD are related to SUD severity in adolescent psychiatric patients.

**Methods:**

We analyzed *N* = 114 self-reports of treatment-seeking German adolescents aged 12 to 18 years, who visited a specialized SUD outpatient unit. Standardized questionnaires were applied to assess SUD severity, the number of TEs and DSM-IV PTSD criteria.

**Results:**

Patients fulfilling PTSD criteria (28% of the total sample) had a higher Drug Use Disorders Identification Test (DUDIT) score compared to non-PTSD patients with TEs (*p* <.001), and compared to adolescents without TEs or PTSD (*p* = .003). Additionally, SUD severity was positively associated with the number of TEs and the number of intrusion, hyperarousal, and avoidance symptoms (all *r* = .33 to.48, all *p* <.01).

**Discussion:**

Adolescent patients with SUD reported 3-times higher rates of TEs, and a 5-time higher prevalence of PTSD following TEs, than the general adolescent population. Adolescent SUD patients with PTSD reported more severe substance use problems than patients without PTSD—regardless of previous TEs. Longitudinal studies are needed in order to investigate the temporal relationship between TEs, PTSD and SUD.

## Introduction

Adolescence, as defined by the World Health Organization, is a phase of life between ages 10 and 19 that marks the transition from childhood to adulthood (1, 2). Adolescents are “biologically wired” (3) to engage in risky and potentially harmful behavior including the use of psychoactive substances, which might lead to the development of a substance use disorder (SUD) (4). Moreover, the increase in risk behavior might lead to identification with substance-related subcultures (e.g. “psychonauts”), in which use of novel psychoactive substances (NPS) is widespread (5). In Germany, the lifetime prevalence rate of SUD in adolescents and young adults is 28.6% (6), with SUD being associated with worse performance in school, worse overall health, higher mortality rates and increased rates of comorbid psychiatric disorders such as post-traumatic stress disorder (PTSD) (7–9).

PTSD is an anxiety disorder that may develop subsequent to exposure to traumatic events (TEs), either experienced or witnessed. TEs are defined as experiences that involve actual or threatened death, serious injury, or threat to the physical integrity of oneself or others, and are responded to with intense fear, helplessness or horror (10). PTSD diagnosis requires an experience of a TE (Criterion A) that is followed by three major symptom clusters: intrusive recollections of the event (Criterion B), avoidance of event-related stimuli, or emotional numbing (Criterion C) and hyperarousal (Criterion D).

Common developmental pathways for SUD and PTSD have been proposed, given the high rates of co-occurrence between the two disorders (11): Firstly, there is evidence from studies in adult patients that SUD and PTSD have common genetic and family environmental risk factors (12). Secondly, substance use in general is related to more frequent risk behavior (13), which increases the likelihood of experiencing TEs, and therefore developing PTSD (14, 15). For example, patients with a SUD are more likely to engage in other-directed than self-directed violence, increasing the risk of encountering TEs (16). Finally, the self-medication hypothesis derives from clinical observations that the specific actions or effects of each class of drugs relieve or change a range of painful affect states in patients with PTSD (11, 17, 18).

Notably, the rate at which people are exposed to TEs peaks during adolescence (19) and this increased exposure is naturally associated with a higher rate of PTSD (14). In sum, adolescents typically engage in high-risk behavior thus increasing their risk for both substance use and TEs, which might lead to subsequent disorders, e.g. SUD and/or PTSD. There is a growing epidemiological and clinical literature documenting the frequent co-occurrence of SUD and PTSD in adolescents (20). Several studies indicate that between 20 and 54% of North American adolescents with SUD also have co-occurring PTSD (21, 22). In German adolescents, SUD occurs at a rate of 30% in patients who fulfill PTSD criteria (23). Moreover, the severity of substance use (except for alcohol use) correlates positively with the number of PTSD symptoms in adolescents (24).

Previous research established the link between SUD and PTSD (11, 18, 20, 24). However, it remains to be explored if SUD severity is only increased in adolescents with co-occurring PTSD, or linked to the occurrence of TEs in general, providing an insight into the pathomechanisms of both disorders.

Here, we assess the prevalence of both lifetime TEs and PTSD according to DSM-IV criteria among German adolescents seeking treatment for a SUD and compare these prevalence rates with the general adolescent population. We compare three groups of SUD treatment-seeking adolescents: adolescents fulfilling DSM-IV PTSD diagnostic criteria (‘PTSD’ group), adolescents with a history of TEs but no PTSD (‘TE’ group) and adolescents with no TEs and no PTSD (‘NoTE’ group).

We hypothesize that (1) adolescents with a SUD present with higher rates of current PTSD and past TEs than the general adolescent population. We predict that (2) SUD severity will be higher in the PTSD and TE group than in the NoTE group and that (3) SUD severity is positively associated with the number of PTSD symptoms present in each symptom cluster.

## Method

### Participants

Between November 2017 and October 2019, *n* = 178 treatment-seeking adolescents at an outpatient clinic for adolescent substance abuse participated in the study. *N* = 64 adolescents (36%) did not complete all necessary questionnaires and were therefore excluded. In the final sample of *n* = 114 patients, the mean age was 15.8 years (*SD* = 1.3) with 43% (*n* = 49) females, see **Table 1**. Secondary education levels were predominantly classified as low level (55%), while household income was predominantly classified as medium level (57%), see **Table 1**.

**Table 1:** Sociodemographic characteristics of the complete sample, and the three subgroups.

*Demographics*	*Total (n* = 114*)*	*Analysis group*			Group comparison		
		*NoTE (n* = 35*)*	*TE (n* = 47*)*	*PTSD (n* = 32*)*	Test statistic (*df*)	p	*Effect size*
Mean age (*SD*)	15.8 (1.3)	15.5 (1.3)	16.0 (1.3)	15.8 (1.2)	*F* (*113*) = 1.32	.271	*η*_p_^2^ = .023
Gender:					χ² (*2*) = 2.427	.297	ϕ = .15
Female (%)	49 (43)	12 (34)	27 (57)	17 (53)			
Male (%)	65 (57)	23 (66)	20 (43)	15 (47)			
Household income: # of patients in category (%), missing *n* = 60	(*n* = 54)	(*n* = 22)	(*n* = 21)	(*n* = 11)	χ² (*4*) = 1.525	.822	.17
Low income	9 (17)	4 (18)	4 (19)	1 (9)			
Middle income	31 (57)	11 (50)	13 (62)	7 (63)			
High income	14 (26)	7 (32)	4 (19)	3 (25)7			
Educational level: # of patients in category (%), missing *n* = 38	(*n* = 76)	(*n* = 22)	(*n* = 34)	(*n* = 20)	χ² (*6*) = 4.773	.573	.25
Low	37(55)	9 (41)	16 (47)	12 (60)			
Middle	20 (26)	6 (27)	11 (32)	3 (15)			
High	8 (11)	4 (18)	3 (9)	1 (5)			
Other	11 (14)	3 (14)	4 (12)	4 (20)			
Substance abuse: # of patients presenting with harmful use or dependence per substance (%), missing *n* = 2	(*n* = 112)	(*n* = 35)	(*n* = 47)	(*n* = 30)			
Alcohol	44 (39)	12 (34)	23 (49)	9 (30)	χ² (*2*) = 3.286	.193	.17
Cannabis	89 (80)	29 (83)	38 (81)	22 (73)	χ² (*2*) = 0.993	.609	.09
Stimulants (amphetamine, methamphetamine, or MDMA)	49 (44)	13 (37)	18 (38)	18 (60)	χ² (*2*) = 4.408	.110	.20

PTSD, Post-traumatic stress disorder according to DSM-IV; TE, Traumatic event; MDMA, methylenedioxymethamphetamine. A clinical psychologist performed the diagnosis of harmful use or dependence syndrome, and multiple diagnoses per patient were possible. For the differences in substance abuse, the p-value was adjusted according to the Bonferroni-procedure to p < .02.

### Procedures

Data collection was embedded into the standard diagnostic procedures at the outpatient clinic. Questionnaires were handed out to the patients and their legal guardians at the first consultation appointment in the outpatient department. The criteria for harmful use and dependence for all relevant psychoactive substances according to the ICD-10 guidelines (25), were assessed in a personal interview by a trained clinical psychologist. Study assessments took place before any intervention started. The study was conducted in accordance with the Declaration of Helsinki. Patients as well as legal guardians were informed about the projects thoroughly and comprehensively. Written informed consent was obtained from all legal guardians. All procedures of this study were approved by the Institutional Review Board of the University Hospital C. G. Carus Dresden (EK 66022018) and registered at clinicaltrials.gov (NCT03444974). No reimbursement was offered to patients.

### Measures

#### Traumatic Experiences, PTSD Symptoms and PTSD Diagnosis

The University of California at Los Angeles Post Traumatic Stress Disorder Reaction Index for DSM-IV (UCLA PTSD (26) is a self-report instrument that screens for exposure to TEs, and assesses PTSD symptoms in school-age children and adolescents. It has been translated for, and used with German-speaking populations (27). The instrument consists of a trauma history section, in which patients indicate the TE that afflicts them the most and the traumatizing features of the event (Criterion A). The next section assesses the frequency of occurrence of PTSD symptoms during the past month (rated from 0 = none of the time to 4 = most of the time). The items map directly onto DSM-IV intrusion/re-experience (Criterion B), avoidance (Criterion C), and hyperarousal (Criterion D) criteria. Scoring algorithms permit tabulation of UCLA PTSD total score, and A, B, C, and D subscale scores. The DSM-IV diagnosis PTSD is given when all four criteria (Criterion A, B, C, and D) are present (26). In the current sample, internal consistency was good for criterion A and B (α = .83 and.86, respectively), and acceptable for criterion C and D (α = .73 and.75, respectively).

#### Substance Use and SUD Severity

The Drug Use Disorders Identification Test [DUDIT; (28)] is a self-report instrument composed of 11 items identifying problems related to substance use. Scoring of the DUDIT is two-fold: items 1 to 9 are scored on a five-point Likert scale, while items 10 and 11 are scored on a three-point scale. The overall score is calculated by summing the scores on all items, with a maximum score of 44. In the current sample, internal consistency of the DUDIT was good with α = .87.

#### SUD Diagnosis

A clinical psychologist assessed criteria for harmful use and dependence syndrome for all relevant psychoactive substances according to ICD-10 (24) in a personal interview with the patients. The SUD diagnosis was established in a consensus-based procedure by interviewing psychologist and a board-certified child and adolescent psychiatrist

#### Sociodemographic Background

Thirty-six generic questions were presented to the parents. We analyzed the questions indicating age in years, gender, and education level (low, medium, high) of the patient as well as yearly household income (low, medium, high). Adolescents’ educational levels and parental income levels were assessed according to previously established criteria (29).

### Analyses

All analyses were conducted with IBM SPSS Statistics 25.0. Based on UCLA PTSD results, patients were separated into three analysis groups: PTSD diagnosis (‘PTSD’ group), TE but no PTSD diagnosis (‘TE’ group), no TE and no PTSD diagnosis (‘NoTE’ group). To investigate the relationship between SUD and PTSD symptoms, Pearson’s correlation coefficient *r* was calculated between DUDIT score, the number of possible TEs, and the number of PTSD symptoms within each criterion cluster (Clusters A, B, C, and D). An analysis of variance (ANOVA) was performed to test for mean differences in DUDIT score between the three groups. To determine if the ANOVA is an appropriate procedure we checked the normality of the dependent variable (DUDIT score) by means of the Shapiro–Wilk test (30). Additionally, to check for homogeneity of variances, Levene’s test was conducted. DUDIT score was normally distributed in the NoTE group (*W* = .944; *p* = .072), the TE group (*W* = .956; *p* = .075), and the PTSD group (*W* = .952; *p* = .16). Further, Levene’s test showed that the variance of DUDIT score was equal across groups, *F* (2, 111) = 0.8, *p* = .452. In cases were the *F*-test across groups was significant, post-hoc multiple comparisons using the Bonferroni correction were conducted to identify groups differing from each other regarding their mean DUDIT score. To detect demographic differences between the groups, χ^2^-tests were conducted. Level of significance was defined as *p* <.05 (two-tailed). Since patients could present with harmful use or dependence for multiple substances, multiple univariate χ^2^-tests were used to assess group differences in this variable. For this specific analysis, the level of significance was adapted according to the Bonferroni-procedure to *p* <.05/3. Effect sizes were classified according to Cohen (31) into small effects (|*r*| ≥.10, partial eta-square η_p_^2^ ≥.01), medium effects (|*r*| ≥.30, η_p_^2^ ≥.06), and large effects (|*r*| ≥.50, η_p_^2^ ≥.14).

## Results

### Sociodemographic Characteristics and Substance Abuse

The majority of patients reported enough symptoms to qualify for cannabis abuse (80%), followed by alcohol abuse (39%) and stimulant (amphetamine, methamphetamine, or MDMA) abuse (44%), see **Table 1**. None of the patients reported use of NPS during the past 12 months. The three groups (PTSD, TE, and NoTE) did not differ in any of the assessed sociodemographic characteristics nor in their SUD diagnoses, see **Table 1**.

### Prevalence Estimates

**Figure 1:**
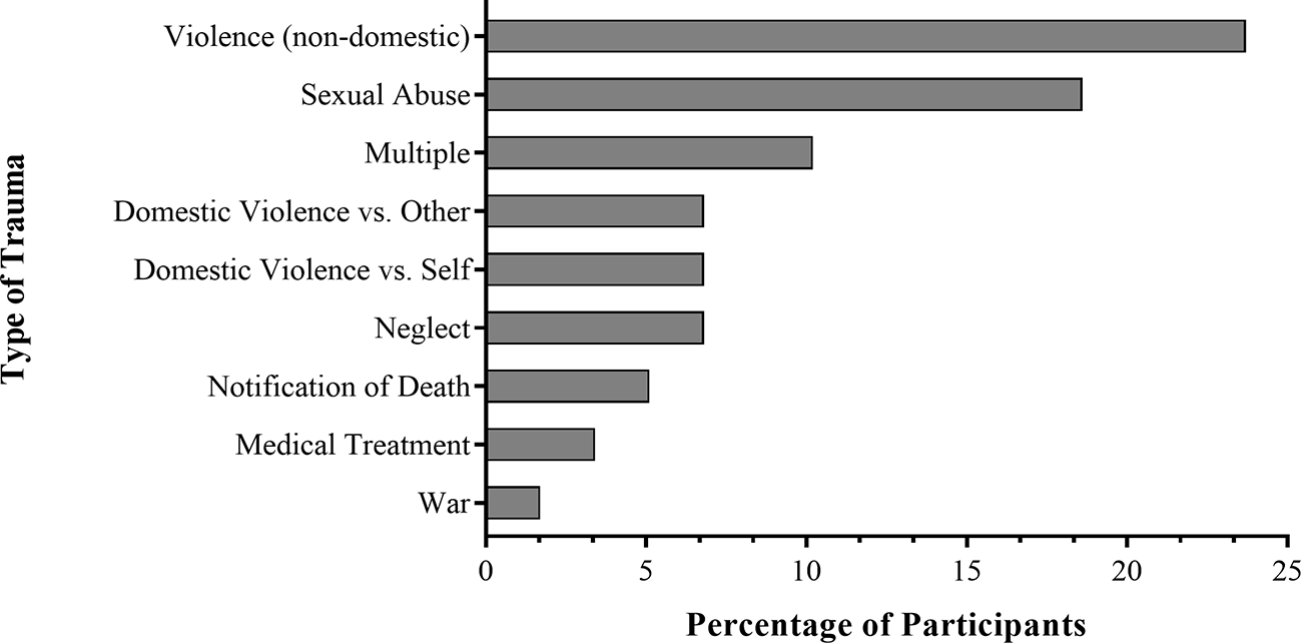
The percentage of patients (*n* = 59) that described a particular type of event as their most traumatizing experience. Total *n* = 114, with *n* = 35 reporting no traumatic experiences, and *n* = 20 not clearly identifying the most traumatizing experience.

The majority of patients (69%) reported at least one lifetime TE. **Figure 1** illustrates that “non-domestic violence” followed by “sexual abuse” were the most-prevalent TEs categories, whereas “war” and “medical treatment” represented the least-frequent categories. Over one third of the patients with TE (41%) also fulfilled the DSM-IV diagnostic criteria for a PTSD according to the UCLA PTSD scale, resulting in a point prevalence of 28% for PTSD in the total sample.

### The Relationship Between TEs, PTSD Symptoms and SUD Severity

The number of symptoms in each PTSD symptom cluster (A = number of traumatizing features of TEs, B = intrusion, C = avoidance, D = hyperarousal) correlated positively with each other (all *r* = .23 to .56, all *p* <.038) and with DUDIT score (all *r* = .33 to.48, all *p* <.003) with an exception of DUDIT score and Cluster A (*r* = .20, *p* = .08). The correlations of DUDIT score and clusters B, C, and D can be classified as medium size effects (*r* =.30 to .49), see **Table 2**.

**Table 2:** Bivariate Pearson correlation coefficients between DUDIT score, the number of Traumatic events (TE), and the number of symptoms present in Clusters A, B, C, and D of the UCLA PTSD Questionnaire.

	DUDIT score	Number of TE	Sum Cluster A	Sum Cluster B	Sum Cluster C	Sum Cluster D
DUDIT score	–	.21*	.20	.33^**^	.35^**^	.48^**^
Number of TE	.21*	–	.13	.34^**^	.27*	.16
Sum Cluster A	.20	.13	–	.45^**^	.27*	.23*
Sum Cluster B	.33^**^	.34^**^	.45^**^	–	.56^**^	.54^**^
Sum Cluster C	.35^**^	.27*	.27*	.56^**^	–	.47^**^
Sum Cluster D	.48^**^	.16	.23*	.54^**^	.47^**^	–

DUDIT, Drug use disorders identification test; PTSD, Post-traumatic stress disorder according to DSM-IV. Cluster A = Traumatizing qualities of event. Cluster B = Intrusion. Cluster C = Avoidance. Cluster D = Hyperarousal. SUD, substance use disorder according to ICD-10, including substance abuse and substance dependence; TE, Traumatic event. *p < 0.05, **p < 0.01.The number of cases per cell varied due to presence of a PTSD between n = 79 and 114.

### Group Differences in SUD Severity

**Figure 2:**
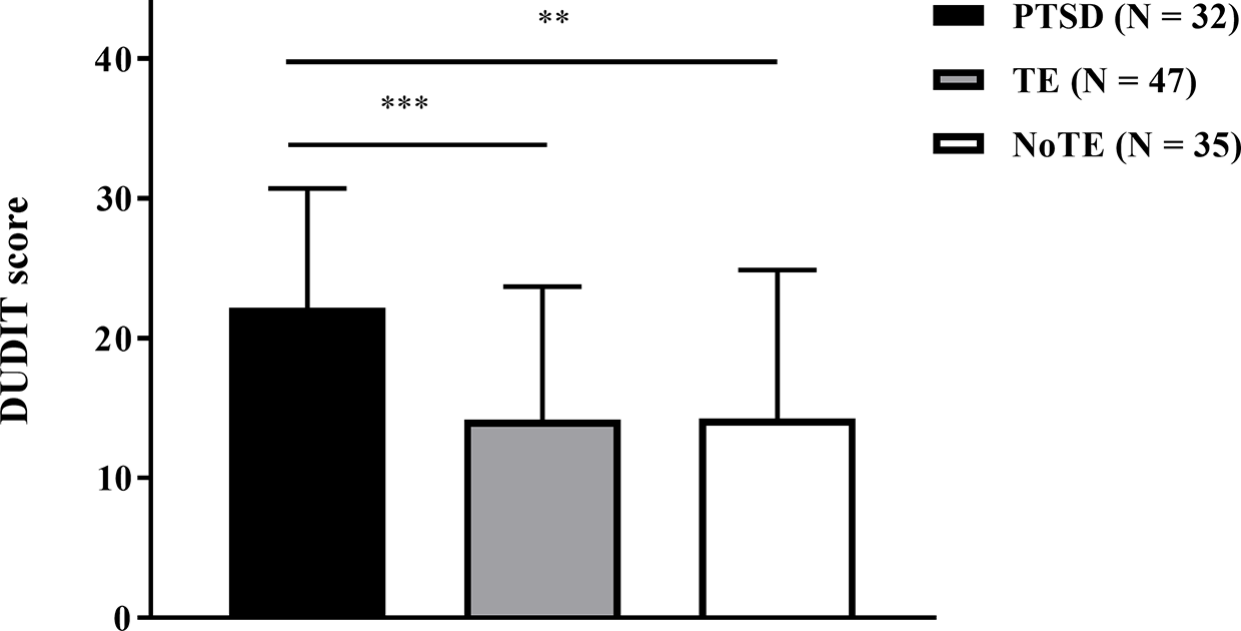
The mean DUDIT score for the PTSD group (*n* = 32), the TE group (*n* = 47), and the NoTE group (*n* = 35). Mean differences were calculated through post-hoc multiple comparisons using the Bonferroni correction (**p < 0.01, ***p < 0.001).

The three patient groups (PTSD, TE, NoTE) differed in their mean DUDIT score, *F* (2, 111) = 7.86, *p* = .001, η_p_^2^ = .124. Post-hoc multiple comparisons revealed that the PTSD group scored a significantly higher mean DUDIT score when compared to the TE group (*t* (77) = 3.812, *p* = .0008), and to the NoTE group (*t* (65) = 3.33, *p* = .003). No difference was found between the TE and the NoTE group (*t* (80) = .039, *p* = .969), see **Figure 2**.

## Discussion

We investigated differences in SUD severity between three groups (PTSD, TE, NoTE) of treatment-seeking adolescents at a German outpatient clinic for adolescent substance abuse. Additionally, we assessed the past-month-prevalence of PTSD and lifetime-prevalence of TE. In accordance with our hypothesis as well as earlier studies, our results indicate that adolescents with SUD were more likely to report TEs [69% vs. 21% (32)], more likely to suffer from PTSD following TEs [41% vs. 8% (33)], and more likely to fulfill the diagnostic criteria for PTSD compared to the general adolescent population [28% vs. 2% (32)]. Contrary to our hypothesis, only the PTSD group displayed more severe SUD symptoms than the TE group and the NoTE group. Additionally, we confirmed the hypothesis that SUD severity is positively associated with the number of hyperarousal, intrusion, and avoidance symptoms.

We were not able to delineate the reason for the positive relationship between the number of PTSD symptoms and SUD severity. Patients with stronger PTSD symptoms might self-medicate more heavily with psychotropic substances, and therefore develop more SUD symptoms (11, 17, 18). Alternatively, when functional substance use develops into a SUD, PTSD symptoms might increase as a results of reduced overall mental health (34). Additionally, the development of SUD-related withdrawal symptoms, such as anxiety or depression, might lead to stronger PTSD symptoms (34). Consequently, self-medication of PTSD symptoms with psychotropic substances should be seen as a risk factor, not only for a subsequent SUD, but also for an increase of PTSD symptoms later on. It remains to be explored, if patients with more severe PTSD engage in more substance use, if patients with heavier substance use are more vulnerable to develop PTSD following TEs, or whether a more complex bidirectional relationship exists. To assess these complex relationships, longitudinal studies are needed that frequently assess occurrence of TEs, PTSD symptoms, substance use, and SUD symptoms.

Our finding that TEs alone are not associated with higher SUD severity is supported by research examining the relationship between PTSD and SUD in adolescents and adults (35–36). Reed et al. (37) found that in adolescents, the presence of PTSD but not TEs without PTSD is associated with a higher risk for future substance abuse or dependence. Furthermore, Driessen et al. (35) observed that a PTSD diagnosis but not the number of TEs correlates with SUD severity. Finally, Kok et al. (36) reported, that trauma-related variables do not add information about variation in drug use severity compared to PTSD symptoms. This line of research indicates that TEs are unrelated to SUD severity and the development of SUD symptoms.

Previous studies with adult SUD patients reported similar results to ours, insofar as all three symptom clusters correlated positively with SUD severity (36, 38). However, research in adolescents indicated only avoidance symptoms being positively associated with SUD severity, whereas hyperarousal symptoms were negatively correlated with SUD severity, and intrusion symptoms were not related to SUD severity at all (24). These conflicting results might be explained by the difference in sample selection. Specifically, we included patients with a SUD regardless of substance, whereas Donbaek et al. (24) excluded patients with an alcohol use disorder (AUD). Thus, the substance of use could be relevant for the relationship between SUD and PTSD symptoms. Indeed, a relationship between the use of specific substances and PTSD symptoms has been observed to exist in adults and adolescents. Explicitly it has been reported that the number of avoidance symptoms is higher in people who use alcohol (11, 38), sedatives, gamma-Hydroxybutyric acid (GHB), opioids (39), and heroin specifically (40). Moreover, people with an alcohol (38) or opioid (39) use disorder present with more hyperarousal symptoms than people who do not. Finally, Khoury et al. (38) and Avant et al. (39) report that the use of cannabis, cocaine, sedatives, and GHB is also related to more intrusion symptoms. While the use of specific substances does not seem to be related to specific symptom clusters, recent findings (11, 24, 38–40) confirm our results, that a higher level of substance use across substances is generally related to a higher number of PTSD symptoms across clusters.

### Limitations

Our sample was limited to treatment-seeking adolescents with a SUD. Therefore, future research could investigate whether the presence of TE or PTSD influences the intensity of recreational substance use as well or if this influence is limited to SUD severity.

Our assessment of TEs and PTSD was based on self-report measures, which might be biased in the sense that patients might not recall all of their TEs. Additionally, all self-report instruments potentially suffer from response bias. Examples include misunderstanding of the items or a social-desirability bias (41). Furthermore, self-reports seem to underestimate trauma-specific cognitions, like intrusion symptoms (42). In future research it might be useful to use additional instruments such as standardized interviews to gain a more accurate picture of the PTSD symptoms clusters and number of TEs.

Furthermore, because we did not collect detailed data on substance use, we could not investigate the relationship between the use of specific substances and the number of PTSD symptoms in each cluster. Additionally, we took no measures to verify self-reports of substance use *via* biological measures. Therefore, even though none of the patients reported use of NPS, they might have unwillingly ingested them instead of a substance they were sold (e.g. NPS in an ecstasy pill).

Finally, our cross-sectional study lacks a possibility to investigate the question of timing and causality concerning PTSD and SUD development. A prospective longitudinal study design is necessary in order to answer the question, which symptoms or disorders develop first, and if TEs precede substance use or vice versa. This line of research would help to clarify the hypotheses concerning the relationship between the two disorders. For example, the finding that substance use and SUD symptoms generally appear after a TE would support the self-medication hypothesis.

### Conclusion

Adolescents with SUD reported 3-time higher rates of TEs, and a 5-time higher prevalence of PTSD following TEs, than the general adolescent population. SUD was more severe in adolescents with PTSD than in adolescents without TEs or with TE anamnesis but no PTSD symptoms. Finally, the level of SUD severity was positively correlated with the number of PTSD-related intrusion, avoidance, and hyperarousal symptoms.

## Data Availability Statement

The datasets generated for this study are available on request to the corresponding author.

## Ethics Statement

The studies involving human participants were reviewed and approved by Institutional Review Board of the University Hospital C. G. Carus, Dresden (EK 66022018). Written informed consent to participate in this study was provided by the participants’ legal guardian/next of kin.

## Author Contributions

LB analyzed the data and wrote the manuscript. SK-P participated in writing the manuscript and data analysis, and contributed to the discussion. VR participated in writing the manuscript and contributed to discussion. YG designed the study, participated in writing the manuscript, and contributed to discussion.

## Funding

The Sächsische Aufbaubank -Förderbank-, (grant 100362999 to YG), funded this study. Open access funding by the Publication Fund of the TU Dresden.

## Conflict of Interest

SK-P received honoraria/fees during the past 12 months: author fees from a publisher of medical books (Mabuse Verlag), and honoraria for one speech from a group of companies (AbbVie Deutschland, Almirall Hermal, Belano medical, Celgene, Janssen-Cilag, LEO Pharma, Lilly Deutschland, Novartis Pharma, Pfizer Pharma, UCB Pharma).

The remaining authors declare that the research was conducted in the absence of any commercial or financial relationships that could be construed as a potential conflict of interest.
